# *N*-(3-Chloro­phen­yl)maleamic acid

**DOI:** 10.1107/S1600536810021446

**Published:** 2010-06-16

**Authors:** B. Thimme Gowda, Miroslav Tokarčík, K. Shakuntala, Jozef Kožíšek, Hartmut Fuess

**Affiliations:** a Department of Chemistry, Mangalore University, Mangalagangotri 574 199, Mangalore, India; b Faculty of Chemical and Food Technology, Slovak Technical University, Radlinského 9, SK-812 37 Bratislava, Slovak Republic; c Institute of Materials Science, Darmstadt University of Technology, Petersenstrasse 23, D-64287 Darmstadt, Germany

## Abstract

In the title compound, C_10_H_8_ClNO_3_, the molecular conformation is stabilized by two intra­molecular hydrogen bonds. The first is a short O—H⋯O hydrogen bond within the maleamic acid unit and the second is a C—H⋯O hydrogen bond which connects the amide group with the phenyl ring. The maleamic acid unit is essentially planar, with an r.m.s. deviation of 0.044 Å, and makes a dihedral angle of 15.2 (1)° with the phenyl ring. In the crystal, inter­molecular N—H⋯O hydrogen bonds link the mol­ecules into *C*(7) chains running [010].

## Related literature

For studies on the effect of ring- and side-chain substitutions on the crystal structures of amides, see: Gowda *et al.* (2010**a* ▶,b* ▶); Prasad *et al.* (2002 ▶); Shakuntala *et al.* (2009 ▶). For hydrogen-bond motifs, see: Bernstein *et al.* (1995 ▶).
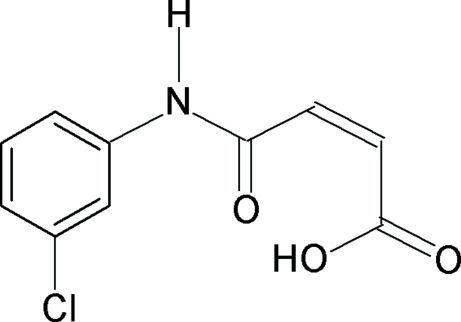


## Experimental

### 

#### Crystal data

C_10_H_8_ClNO_3_
*M* *_r_* = 225.62
Monoclinic, 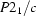

*a* = 10.7779 (3) Å
*b* = 13.2103 (4) Å
*c* = 7.1372 (2) Å
β = 104.976 (3)°
*V* = 981.69 (5) Å^3^
*Z* = 4
Mo *K*α radiation
μ = 0.37 mm^−1^
*T* = 295 K
0.55 × 0.09 × 0.06 mm

#### Data collection

Oxford Diffraction Gemini R, CCD diffractometer
Absorption correction: analytical (*CrysAlis PRO*; Oxford Diffraction, 2009 ▶) *T*_min_ = 0.852, *T*_max_ = 0.982
15632 measured reflections
1829 independent reflections
1533 reflections with *I* > 2σ(*I*)
*R*_int_ = 0.027

#### Refinement

*R*[*F* ^2^ > 2σ(*F* ^2^)] = 0.028
*wR*(*F* ^2^) = 0.081
*S* = 1.08
1829 reflections
136 parameters
H-atom parameters constrained
Δρ_max_ = 0.16 e Å^−3^
Δρ_min_ = −0.16 e Å^−3^

### 

Data collection: *CrysAlis PRO* (Oxford Diffraction, 2009 ▶); cell refinement: *CrysAlis PRO*; data reduction: *CrysAlis PRO*; program(s) used to solve structure: *SHELXS97* (Sheldrick, 2008 ▶); program(s) used to refine structure: *SHELXL97* (Sheldrick, 2008 ▶); molecular graphics: *ORTEP-3* (Farrugia, 1997 ▶) and *DIAMOND* (Brandenburg, 2002 ▶); software used to prepare material for publication: *SHELXL97*, *PLATON* (Spek, 2009 ▶) and *WinGX* (Farrugia, 1999 ▶).

## Supplementary Material

Crystal structure: contains datablocks I, global. DOI: 10.1107/S1600536810021446/bx2280sup1.cif

Structure factors: contains datablocks I. DOI: 10.1107/S1600536810021446/bx2280Isup2.hkl

Additional supplementary materials:  crystallographic information; 3D view; checkCIF report

## Figures and Tables

**Table 1:** Hydrogen-bond geometry (Å, °)

*D*—H⋯*A*	*D*—H	H⋯*A*	*D*⋯*A*	*D*—H⋯*A*
O2—H2*A*⋯O1	0.90	1.60	2.4992 (14)	176
N1—H1*N*⋯O3^i^	0.86	1.99	2.8403 (15)	172
C6—H6⋯O1	0.93	2.31	2.8658 (16)	118

Symmetry code: (i) 
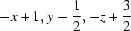
.

**Fig. 1.**
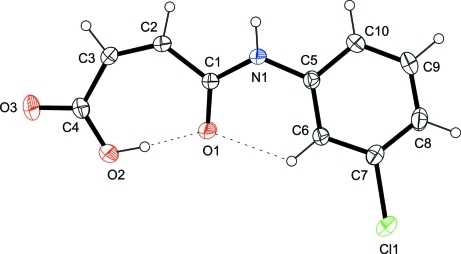
Molecular structure of (I) showing the atom labelling scheme. Displacement ellipsoids are drawn at the 30% probability level. H atoms are represented as small spheres of arbitrary radii and a short intramolecular O—H···O bond as dashed line.

**Fig. 2.**
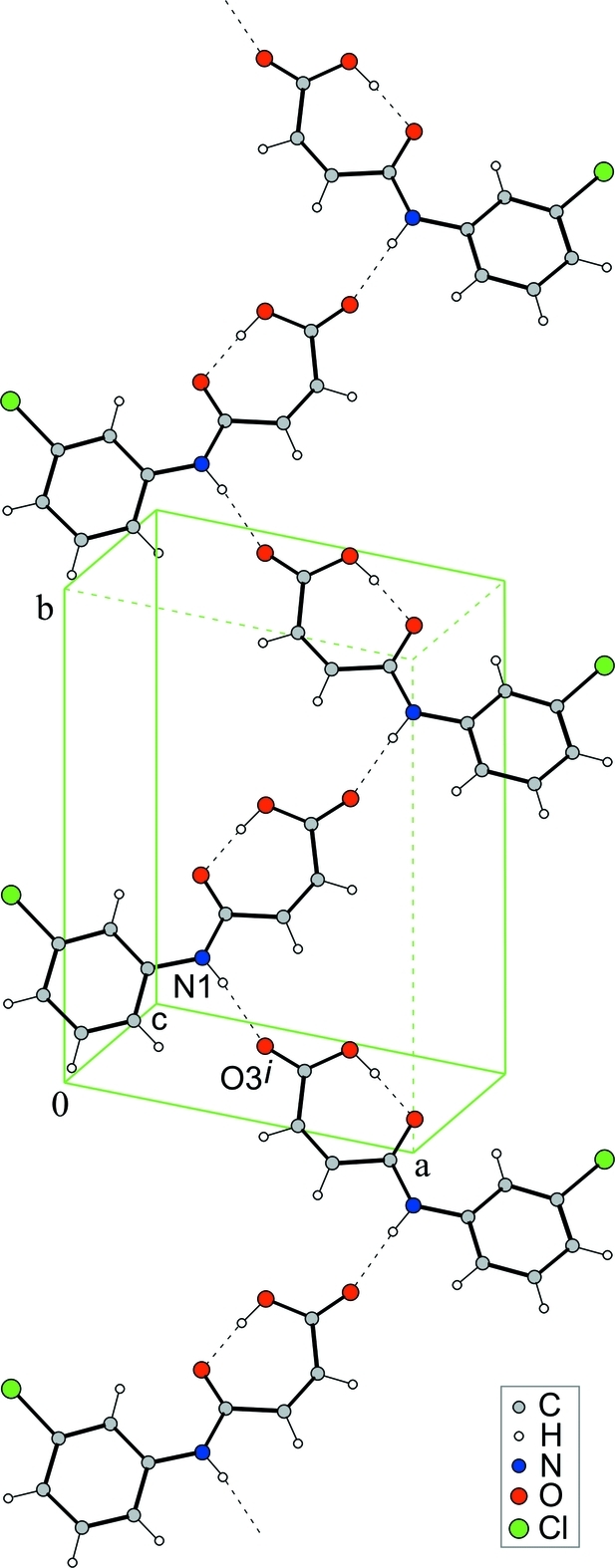
Part of crystal structure of (I) showing one-dimensional chain of molecules extending along the [0 1 0] direction and linked by intermolecular N–H···O hydrogen bonds. Hydrogen bonds are shown as dashed lines. Symmetry code (i): -x + 1, y - 1/2, -z + 3/2.

## supplementary crystallographic information

### Comment

As a part of studying the effect of ring and side chain substitutions on the crystal structures of biologically significant amides (Gowda *et al.*, 2010*a,b*; Prasad *et al.*, 2002; Shakuntala *et al.*, 2009), the crystal structure of *N*-(3-chlorophenyl)maleamic acid (I) has been determined (Fig. 1).The conformations of the N—H and the C=O bonds in the amide segment are *anti* to each other. The conformation of the N—H bond is also *anti* to the *meta*-Cl group in the phenyl ring.In the maleamic acid moiety, the amide C=O bond is *anti* to the adjacent C—H bond, while the carboxyl C=O bond is *syn* to the adjacent C—H bond. The observed rare *anti* conformation of the C=O and O—H bonds of the acid group is similar to that observed in *N*-(2-methylphenyl)-maleamic acid (Gowda *et al.*, 2010*b*), *N*-(2,5-dichlorophenyl)-maleamic acid (Shakuntala *et al.*, 2009) and *N*-(3,5-dichlorophenyl)- maleamic acid (Gowda *et al.*, 2010*a*).

The molecular structure of (I) is stabilized by two intramolecular hydrogen bonds (Figure 1): the first is a short O—H···O hydrogen bond within maleamic acid unit and the second is a C—H···O hydrogen bond which connects the amide group with the phenyl ring. Amidic O1 atom acts as bifurcated acceptor of O2—H2A···O1 and C6—H6···O1 intramolecular hydrogen bonds (Table 1).The maleamic acid unit is essentially planar, with r.m.s.deviation of 0.044 Å and makes dihedral angle of 15.2 (1)° with the phenyl ring. The torsion angle C1—N1—C5—C6 = -17.6 (2)° defines the orientation of the phenyl ring towards the central amide group –NHCO. The molecular structure is stabilized by two types intramolecular C—H···O and O—H···O interactions with H···O distances of 1.60 and 2.31 Å respectively and one intermolecular N—H···O hydrogen bonds link the molecules into chains with graph-set notation C(7) (Bernstein *et al.*, 1995) running along the [0 1 0] direction, Table 1, Figure 2.

### Experimental

The solution of maleic anhydride (0.025 mol) in toluene (25 ml) was treated dropwise with the solution of 3-chloroaniline (0.025 mol) also in toluene (20 ml) with constant stirring. The resulting mixture was warmed with stirring for over 30 min and set aside for an additional 30 min at room temperature for completion of the reaction. The mixture was then treated with dilute hydrochloric acid to remove the unreacted 3-chloroaniline. The resultant solid *N*-(3-chlorophenyl)maleamic acid was filtered under suction and washed thoroughly with water to remove the unreacted maleic anhydride and maleic acid. It was recrystallized to constant melting point from ethanol. The purity of the compound was checked by elemental analysis and characterized by its infrared spectra.

Rod like colourless single crystals used in X-ray diffraction studies were grown in an ethanol solution by slow evaporation at room temperature.

### Refinement

All H atoms were found in difference maps and further treated as riding atoms with C—H = 0.93 Å, N—H = 0.86Å and O—H = 0.90 Å. The *U*_iso_(H) values were set at 1.2*U*_eq_(C aromatic, N) and 1.5*U*_eq_(O).

### Crystal data

**Table:**

C_10_H_8_ClNO_3_	*F*(000) = 464
*M**_r_* = 225.62	*D*_x_ = 1.527 Mg m^−^^3^
Monoclinic, *P*2_1_/*c*	Mo *K*α radiation, λ = 0.71073 Å
Hall symbol: -P 2ybc	Cell parameters from 8764 reflections
*a* = 10.7779 (3) Å	θ = 2.0–29.5°
*b* = 13.2103 (4) Å	µ = 0.37 mm^−^^1^
*c* = 7.1372 (2) Å	*T* = 295 K
β = 104.976 (3)°	Rod, colourless
*V* = 981.69 (5) Å^3^	0.55 × 0.09 × 0.06 mm
*Z* = 4	

### Data collection

**Table:**

Oxford Diffraction Gemini R, CCD diffractometer	1829 independent reflections
graphite	1533 reflections with *I* > 2σ(*I*)
Detector resolution: 10.434 pixels mm^-1^	*R*_int_ = 0.027
ω scans	θ_max_ = 25.5°, θ_min_ = 2.0°
Absorption correction: analytical (*CrysAlis PRO*; Oxford Diffraction, 2009)	*h* = −13→13
*T*_min_ = 0.852, *T*_max_ = 0.982	*k* = −16→16
15632 measured reflections	*l* = −8→8

### Refinement

**Table:**

Refinement on *F*^2^	Primary atom site location: structure-invariant direct methods
Least-squares matrix: full	Secondary atom site location: difference Fourier map
*R*[*F*^2^ > 2σ(*F*^2^)] = 0.028	Hydrogen site location: inferred from neighbouring sites
*wR*(*F*^2^) = 0.081	H-atom parameters constrained
*S* = 1.08	*w* = 1/[σ^2^(*F*_o_^2^) + (0.0488*P*)^2^ + 0.0776*P*] where *P* = (*F*_o_^2^ + 2*F*_c_^2^)/3
1829 reflections	(Δ/σ)_max_ = 0.001
136 parameters	Δρ_max_ = 0.16 e Å^−^^3^
0 restraints	Δρ_min_ = −0.16 e Å^−^^3^

### Special details

**Table:**

Geometry. All e.s.d.'s (except the e.s.d. in the dihedral angle between two l.s. planes) are estimated using the full covariance matrix. The cell e.s.d.'s are taken into account individually in the estimation of e.s.d.'s in distances, angles and torsion angles; correlations between e.s.d.'s in cell parameters are only used when they are defined by crystal symmetry. An approximate (isotropic) treatment of cell e.s.d.'s is used for estimating e.s.d.'s involving l.s. planes.
Refinement. Refinement of *F*^2^ against ALL reflections. The weighted *R*-factor *wR* and goodness of fit *S* are based on *F*^2^, conventional *R*-factors *R* are based on *F*, with *F* set to zero for negative *F*^2^. The threshold expression of *F*^2^ > σ(*F*^2^) is used only for calculating *R*-factors(gt) *etc*. and is not relevant to the choice of reflections for refinement. *R*-factors based on *F*^2^ are statistically about twice as large as those based on *F*, and *R*- factors based on ALL data will be even larger.

### Fractional atomic coordinates and isotropic or equivalent isotropic displacement parameters (Å^2^)

**Table:**

	*x*	*y*	*z*	*U*_iso_*/*U*_eq_	
C1	0.31064 (12)	0.29347 (9)	0.57205 (19)	0.0320 (3)	
C2	0.44462 (13)	0.28772 (10)	0.6940 (2)	0.0372 (3)	
H2	0.4774	0.2228	0.7228	0.045*	
C3	0.52416 (13)	0.36307 (11)	0.7676 (2)	0.0401 (3)	
H3	0.6043	0.3415	0.8403	0.048*	
C4	0.50988 (14)	0.47472 (11)	0.7564 (2)	0.0426 (4)	
C5	0.12674 (13)	0.18104 (10)	0.42427 (19)	0.0319 (3)	
C6	0.03012 (12)	0.25287 (10)	0.38134 (18)	0.0328 (3)	
H6	0.0463	0.3197	0.4214	0.039*	
C7	−0.09097 (13)	0.22302 (11)	0.27750 (19)	0.0367 (3)	
C8	−0.11862 (14)	0.12464 (12)	0.2179 (2)	0.0444 (4)	
H8	−0.201	0.1061	0.149	0.053*	
C9	−0.02125 (16)	0.05452 (12)	0.2627 (2)	0.0509 (4)	
H9	−0.0383	−0.0123	0.2236	0.061*	
C10	0.10121 (14)	0.08138 (11)	0.3646 (2)	0.0432 (4)	
H10	0.1662	0.0331	0.3932	0.052*	
N1	0.25350 (10)	0.20331 (8)	0.53348 (16)	0.0346 (3)	
H1N	0.2998	0.1519	0.5813	0.042*	
O1	0.25666 (9)	0.37411 (7)	0.51038 (16)	0.0479 (3)	
O2	0.40462 (10)	0.51612 (8)	0.65145 (18)	0.0565 (3)	
H2A	0.3485	0.4673	0.5973	0.085*	
O3	0.59801 (11)	0.52618 (9)	0.84566 (19)	0.0666 (4)	
Cl1	−0.21185 (3)	0.31393 (3)	0.22262 (6)	0.05280 (16)	

### Atomic displacement parameters (Å^2^)

**Table:**

	*U*^11^	*U*^22^	*U*^33^	*U*^12^	*U*^13^	*U*^23^
C1	0.0286 (7)	0.0273 (7)	0.0383 (7)	0.0013 (5)	0.0054 (6)	0.0006 (6)
C2	0.0316 (7)	0.0292 (7)	0.0465 (8)	0.0040 (6)	0.0023 (6)	0.0016 (6)
C3	0.0284 (7)	0.0370 (8)	0.0484 (8)	0.0011 (6)	−0.0016 (6)	0.0001 (6)
C4	0.0387 (8)	0.0345 (8)	0.0524 (9)	−0.0072 (6)	0.0080 (7)	−0.0053 (6)
C5	0.0306 (7)	0.0300 (7)	0.0336 (7)	−0.0038 (5)	0.0054 (6)	0.0015 (5)
C6	0.0314 (7)	0.0286 (7)	0.0358 (7)	−0.0026 (6)	0.0042 (6)	0.0003 (5)
C7	0.0307 (7)	0.0427 (8)	0.0344 (7)	−0.0021 (6)	0.0045 (6)	0.0031 (6)
C8	0.0362 (8)	0.0462 (9)	0.0461 (8)	−0.0128 (7)	0.0019 (6)	−0.0033 (7)
C9	0.0523 (10)	0.0333 (8)	0.0626 (10)	−0.0119 (7)	0.0069 (8)	−0.0096 (7)
C10	0.0411 (8)	0.0294 (7)	0.0560 (9)	−0.0008 (6)	0.0070 (7)	−0.0021 (6)
N1	0.0293 (6)	0.0257 (6)	0.0444 (7)	0.0022 (5)	0.0014 (5)	0.0023 (5)
O1	0.0340 (5)	0.0281 (5)	0.0707 (7)	0.0001 (4)	−0.0058 (5)	0.0070 (5)
O2	0.0445 (6)	0.0282 (6)	0.0871 (9)	−0.0008 (5)	−0.0004 (6)	−0.0016 (5)
O3	0.0538 (7)	0.0450 (7)	0.0886 (9)	−0.0191 (6)	−0.0038 (6)	−0.0113 (6)
Cl1	0.0326 (2)	0.0540 (3)	0.0625 (3)	0.00544 (16)	−0.00438 (17)	0.00293 (18)

### Geometric parameters (Å, °)

**Table:**

C1—O1	1.2389 (16)	C6—C7	1.3813 (18)
C1—N1	1.3364 (17)	C6—H6	0.93
C1—C2	1.4828 (19)	C7—C8	1.376 (2)
C2—C3	1.330 (2)	C7—Cl1	1.7404 (15)
C2—H2	0.93	C8—C9	1.374 (2)
C3—C4	1.483 (2)	C8—H8	0.93
C3—H3	0.93	C9—C10	1.379 (2)
C4—O3	1.2073 (18)	C9—H9	0.93
C4—O2	1.3069 (18)	C10—H10	0.93
C5—C6	1.3834 (19)	N1—H1N	0.86
C5—C10	1.3891 (19)	O2—H2A	0.90
C5—N1	1.4178 (17)		
			
O1—C1—N1	122.96 (12)	C5—C6—H6	120.8
O1—C1—C2	123.32 (12)	C8—C7—C6	122.32 (13)
N1—C1—C2	113.72 (11)	C8—C7—Cl1	119.50 (11)
C3—C2—C1	128.61 (13)	C6—C7—Cl1	118.19 (11)
C3—C2—H2	115.7	C9—C8—C7	118.25 (13)
C1—C2—H2	115.7	C9—C8—H8	120.9
C2—C3—C4	132.50 (13)	C7—C8—H8	120.9
C2—C3—H3	113.7	C8—C9—C10	121.25 (14)
C4—C3—H3	113.7	C8—C9—H9	119.4
O3—C4—O2	120.99 (14)	C10—C9—H9	119.4
O3—C4—C3	118.36 (14)	C9—C10—C5	119.50 (14)
O2—C4—C3	120.65 (13)	C9—C10—H10	120.2
C6—C5—C10	120.27 (12)	C5—C10—H10	120.2
C6—C5—N1	122.87 (11)	C1—N1—C5	128.71 (11)
C10—C5—N1	116.85 (12)	C1—N1—H1N	115.6
C7—C6—C5	118.41 (12)	C5—N1—H1N	115.6
C7—C6—H6	120.8	C4—O2—H2A	109.5
			
O1—C1—C2—C3	5.1 (2)	Cl1—C7—C8—C9	179.82 (12)
N1—C1—C2—C3	−175.30 (14)	C7—C8—C9—C10	0.0 (2)
C1—C2—C3—C4	0.0 (3)	C8—C9—C10—C5	0.4 (2)
C2—C3—C4—O3	177.02 (16)	C6—C5—C10—C9	−0.2 (2)
C2—C3—C4—O2	−3.3 (3)	N1—C5—C10—C9	178.16 (13)
C10—C5—C6—C7	−0.4 (2)	O1—C1—N1—C5	−1.2 (2)
N1—C5—C6—C7	−178.62 (12)	C2—C1—N1—C5	179.26 (12)
C5—C6—C7—C8	0.8 (2)	C6—C5—N1—C1	−17.6 (2)
C5—C6—C7—Cl1	−179.60 (10)	C10—C5—N1—C1	164.11 (13)
C6—C7—C8—C9	−0.5 (2)		

### Hydrogen-bond geometry (Å, °)

**Table:**

*D*—H···*A*	*D*—H	H···*A*	*D*···*A*	*D*—H···*A*
O2—H2A···O1	0.90	1.60	2.4992 (14)	176
N1—H1N···O3^i^	0.86	1.99	2.8403 (15)	172
C6—H6···O1	0.93	2.31	2.8658 (16)	118

Symmetry codes: (i) −*x*+1, *y*−1/2, −*z*+3/2.

## References

[bb1] Bernstein, J., Davis, R. E., Shimoni, L. & Chang, N.-L. (1995). *Angew. Chem. Int. Ed. Engl.* **34**, 1555–1573.

[bb2] Brandenburg, K. (2002). *DIAMOND* Crystal Impact GbR, Bonn, Germany.

[bb3] Farrugia, L. J. (1997). *J. Appl. Cryst.* **30**, 565.

[bb4] Farrugia, L. J. (1999). *J. Appl. Cryst.* **32**, 837–838.

[bb5] Gowda, B. T., Tokarčík, M., Kožíšek, J., Shakuntala, K. & Fuess, H. (2010*a*). *Acta Cryst.* E**66**, o51. 10.1107/S1600536809051484 PMC2980102 21580154

[bb6] Gowda, B. T., Tokarčík, M., Shakuntala, K., Kožíšek, J. & Fuess, H. (2010*b*). *Acta Cryst.* E**66**, o1554. 10.1107/S160053681002012X PMC3006921 21587799

[bb7] Oxford Diffraction (2009). *CrysAlis PRO* Oxford Diffraction Ltd, Yarnton, England.

[bb8] Prasad, S. M., Sinha, R. B. P., Mandal, D. K. & Rani, A. (2002). *Acta Cryst.* E**58**, o1296–o1297.

[bb9] Shakuntala, K., Gowda, B. T., Tokarčík, M. & Kožíšek, J. (2009). *Acta Cryst.* E**65**, o3119. 10.1107/S1600536809048715 PMC2972031 21578844

[bb10] Sheldrick, G. M. (2008). *Acta Cryst.* A**64**, 112–122. 10.1107/S0108767307043930 18156677

[bb11] Spek, A. L. (2009). *Acta Cryst.* D**65**, 148–155. 10.1107/S090744490804362X PMC2631630 19171970

